# Apatinib preferentially inhibits PC9 gefitinib-resistant cancer cells by inducing cell cycle arrest and inhibiting VEGFR signaling pathway

**DOI:** 10.1186/s12935-019-0836-8

**Published:** 2019-05-02

**Authors:** Yong-An Song, Ting Ma, Xue-Yan Zhang, Xiang-Song Cheng, Ayobami-Matthew Olajuyin, Zhi-Fu Sun, Xiao-Ju Zhang

**Affiliations:** 1 The People’s Hospital of Zhengzhou University, Zhengzhou, 450000 China; 2 Key Laboratory of Technology of Drug Preparation, Zhengzhou University, Zhengzhou, China; 3 School of Pharmaceutical Sciences, Zhengzhou University, Zhengzhou, 450001 People’s Republic of China; 4 Department of Health Sciences Research, Mayo Clinic, Rochester, MN 55905 USA

**Keywords:** Apatinib, PC9 cells, Gefitinib-resistance, Cell cycle arrest, VEGF2 pathway

## Abstract

**Background:**

Lung cancer is one of the most common and deadly tumors around the world. Targeted therapy for patients with certain mutations, especially by use of tyrosine kinase inhibitors (TKIs) targeting epidermal growth factor receptor (EGFR), has provided significant benefit to patients. However, gradually developed resistance to the therapy becomes a major challenge in clinical practice and an alternative to treat such patients is needed. Herein, we report that apatinib, a novel anti-angiogenic drug, effectively inhibits obtained gefitinib-resistant cancer cells but has no much effect on their parental sensitive cells.

**Methods:**

Gefitinib-resistant lung cancer cell line (PC9GR) was established from its parental sensitive line (PC9) with a traditional EGFR mutation after long time exposure to gefitinib. Different concentrations of apatinib were used to treat PC9, PC9GR, and other two lung cancer cell lines for its anti-growth effects. RNA sequencing was performed on PC9, PC9GR, and both after apatinib treatment to detect differentially expressed genes and involved pathways. Protein expression of key cycle regulators p57, p27, CDK2, cyclin E2, and pRb was detected using Western blot. Xenograft mouse model was used to assess the anti-tumor activity of apatinib in vivo.

**Results:**

The established PC9GR cells had over 250-fold increased resistance to gefitinib than its sensitive parental PC9 cells (IC_50_ 5.311 ± 0.455 μM vs. 0.020 ± 0.003 μM). The PC9GR resistance cells obtained the well-known T790M mutation. Apatinib demonstrated much stronger ( ~ fivefold) growth inhibition on PC9GR cells than on PC9 and other two lung cancer cell lines, A549 and H460. This inhibition was mostly achieved through cell cycle arrest of PC9GR cells in G1 phase. RNA-seq revealed multiple changed pathways in PC9GR cells compared to the PC9 cells and after apatinib treatment the most changed pathways were cell cycle and DNA replication where most of gene activities were repressed. Consistently, protein expression of p57, CDK2, cyclin E2, and pRb was significantly impacted by apatinib in PC9GR cells. Oral intake of apatinib in mouse model significantly inhibited establishment and growth of PC9GR implanted tumors compared to PC9 established tumors. VEGFR2 phosphorylation in PC9GR tumors after apatinib treatment was significantly reduced along with micro-vessel formation.

**Conclusions:**

Apatinib demonstrated strong anti-proliferation and anti-growth effects on gefitinib resistant lung cancer cells but not its parental sensitive cells. The anti-tumor effect was mostly due to apatinib induced cell cycle arrest and VEGFR signaling pathway inhibition. These data suggested that apatinib may provide a benefit to patients with acquired resistance to EGFR-TKI treatment.

**Electronic supplementary material:**

The online version of this article (10.1186/s12935-019-0836-8) contains supplementary material, which is available to authorized users.

## Background

Lung cancer is the leading cause of cancer deaths around the world, with 5-year survival rate of no more than 15% for all of the stages combined. Non-small cell lung cancer (NSCLC) represents approximately 85% of all lung cancer cases [1, 2]. Traditional therapeutic strategies—chemotherapy and radiotherapy—are associated with unsatisfying outcomes, exacerbated by the difficulties of early detection [3]. Due to the toxic side effects and low efficacy of chemotherapeutic drugs, molecular-targeted therapy has emerged as a more effective treatment modality for patients with lung cancer [4]. Alterations of epidermal growth factor receptor (EGFR) have been identified in a variety of human tumors, including lung, breast, head and neck, and ovarian cancers [5]. Activated EGFR with mutations has been reported to promote cell survival, proliferation, invasion, and metastasis through activation of Janus kinase/signal transducers and activators of transcription (JAK/STAT), phosphoinositol 3-kinase (PI3K)/Akt, and mitogen-activated protein kinase (MAPK) pathways [6, 7]. These observations have established EGFR as a target for cancer therapy [8]. Data have shown that patients with NSCLC harboring EGFR-activated mutations exhibit a dramatic tumor regression from EGFR tyrosine kinase inhibitors (TKIs), such as gefitinib and erlotinib [9–13]. However, these inhibitors are universally limited by the development of acquired drug resistance [14–16], which often is developed after 8–16 months of treatment [17, 18]. The most common resistance mechanism is subsequent EGFR T790M mutation, with acquired resistance to either gefitinib or erlotinib, detected in up to 60% of EGFR-mutant NSCLC patients [19–21]. In spite of clearly demonstrated benefits to NSCLC patients with TKI-sensitive EGFR mutations, acquired drug resistance is a significant clinical challenge in EGFR-TKI treatment [22, 23]. Therefore, understanding of the resistance mechanisms and finding an alternative treatment strategy are critical in patient care.

Apatinib is a novel and highly selective inhibitor of the vascular endothelial growth factor receptor 2 (VEGFR2), which blocks the downstream signal transduction of VEGFR2 at the cellular level [18]. Therapeutic strategies against VEGFR have been studied extensively due to the important roles of VEGFR in carcinogenesis [24–26]. These strategies include the use of extracellular inhibitors, such as neutralization antibodies, against VEGF/VEGFR [27] and intracellular inhibitors such as small-molecule inhibitors against VEGFR [28]. Apatinib has been found to exert a promising anti-tumor activity in various cancers [29, 30]. In clinical trials, apatinib has achieved good results in various types of tumor, such as metastatic gastric cancer [30, 31], metastatic breast cancer [29, 32], and advanced NSCLC [33].

The EGFR-mutant PC9 cell line has been used as a model of drug resistance following prolonged exposure to gefitinib or other reversible EGFR kinase inhibitors in vitro [34–36]. After continued exposure of gefitinib, we successfully established PC9 gefitinib-resistant cells (PC9GR cells). By drug screening, we accidently found that unlike its parental cell line this resistant PC9GR cell line was highly sensitive to apatinib treatment. We further explored underlying molecular mechanisms and tested its effects on in-vivo mouse models. Our data showed that apatinib had strong anti-tumor effects on gefitinib-resistant tumors and may be a potential drug for patients with acquired resistance to TKIs.

## Materials and methods

### Lung cancer cell lines and reagents

Human lung cancer cells A549, H460, and H1650 (obtained from Cell Resource Center of Shanghai Institutes for Biological Sciences, Chinese Academy of Sciences) and PC9 cells (presented by Professor Yilong Wu from Zhongshan University) were maintained in RPMI-1640 complete medium (BI, Israel) containing 10% FBS (5% CO_2_, 37 °C) and cultured according to the protocol.

Apatinib and gefitinib (Selleck Chemicals, Houston, USA) were dissolved in DMSO to make a stock solution. Working concentrations were created by diluting the stock solution in RPMI-1640 media containing 10% fetal bovine serum (BI, Israel). Reactive oxygen test kits were purchased from Beyotime Biotechnology Co., Ltd. (Nantong, People's Republic of China). The primary antibodies against p57, p27, cyclin E2, CDK2, VEGFR2, GAPDH, Phospho-Rb (Ser807/811), and Phospho-VEGF Receptor 2 (Tyr951) were purchased from Cell Signaling Technology, Inc. (Danvers, MA, USA).

### Cell proliferation assays

#### Viable cell counting by colorimetric assay

The effect of apatinib on cell proliferation was assessed by counting viable cells using a colorimetric assay in the Cell Counting Kit-8 (CCK8, Solarbio, China). Cells were seeded in 96-well plates at 3 × 10^3^/per well for 24 h before apatinib or gefitinib were added for 72 h. The concentrations of apatinib were from 0 to 32 μM (twofold diluted) and the concentrations of gefitinib (twofold diluted) were from 0 to 64 μM. The absorbance at 450 nm was measured by the spectrophotometer. The percent cytotoxicity was calculated using the following formula: % cytotoxicity = [1  − (absorbance of the experimental well − absorbance of the blank) / (absorbance of the untreated control well − absorbance of the blank)] × 100. The drug concentration required to inhibit cell growth by 50% (IC_50_) was determined from concentration–response curves created with SPSS19.0 software. The results are reported as the mean ± standard deviation (SD) of three independent experiments. The cell viability curves at different concentrations were created with GraphPad Prism 6.0 software.

#### Cell proliferation by CFDA-SE

Cell proliferation was further determined using CFDA-SE cell tracer kit (Beyotime, Haimen, China) in accordance with the manufacturer’s instructions. CFDA-SE can penetrate the cell membrane and enter the cell to be catalytically decomposed into CFSE by intracellular esterase. CFSE can occasionally and irreversibly bind to lysine residues or other amino groups of intracellular proteins, and then CFSE labels these proteins and evenly distributes them to the daughter cells after division. PC9 and PC9GR cells were labeled with CFDA-SE and then seeded on 6-well plates. After 24 h of incubation, the medium was replaced with fresh medium containing different concentrations of apatinib. The cells were harvested after 24 h and then washed twice with phosphate buffered solution (PBS). The fluorescence intensity was measured by flow cytometry (BD Biosciences, San Jose, CA, USA).

#### Colony forming assay

PC9 or PC9GR cells (2000 cells/well) were seeded in a 6-well plate and incubated for 24 h, then the media were replaced with fresh media containing different concentrations of apatinib (0, 0.5, 1, 2, and 4 μM). After 7 days of treatment, the cells were washed twice with PBS, fixed with 4% paraformaldehyde, and colonies were visualized using 0.1% crystal violet staining. The cells were imaged, and the number of colonies was quantified by ImageJ software (National Institutes of Health). A group of > 10 cells was defined as one colony. All of the experiments were performed in triplicate per treatment condition.

### Apoptosis analysis

PC9 and PC9GR cells that underwent apoptosis were analyzed using Annexin V-FITC/propidium iodide (PI) Apoptosis Detection Kit (KeyGEN BioTECH, Nanjing, China) according to the manufacturer’s instructions, via flow cytometry. In brief, PC9 or PC9GR cells were seeded in 6-well plates (2.0 × 10^6^ cells/well) and incubated overnight, then treated with various concentrations of apatinib for additional 48 h. Cells were harvested and washed twice with cold PBS and then resuspended in 500 μL binding buffer containing 5 μL Annexin V-FITC staining solution. Next, 5 μL PI staining solution was added to the cell suspension. The cells were incubated in the dark for 30 min. Cells were then analyzed via flow cytometry (488 nm excitation and 600 nm emission filters) using a BD FACS Calibur flow cytometer (BD FACS Canto™, BD Biosciences). The apoptotic cells were identified by the localization of Annexin V and PI.

### Mitochondrial membrane potential analysis

The mitochondrial membrane potential (MMP) was determined using a JC-1 assay (Beyotime Institute of Biotechnology, China) according to the manufacturer’s instructions. PC9 or PC9GR cells were plated in 6-well plates (2.0 × 10^6^ cells/well) and incubated for 24 h, then cultured with different concentrations of apatinib for another 24 h. After incubation, cells were harvested with trypsin, washed twice with PBS, supplemented with 500 μL JC-1 dye staining solution, and then incubated at 37 °C for 30 min in the dark. After incubation, the cells were centrifuged at 400*g* × 5 min and washed twice with 1× incubation buffer. The cells were then resuspended in 500 μL 1× incubation buffer and fluorescence was detected using flow cytometry (488 nm excitation and 525 nm emission filters).

### Cell cycle analysis

PC9 or PC9GR cells were harvested 48 h after treatment with different concentrations of apatinib and washed twice with ice-cold PBS. The cells were fixed and permeabilized with 70% ice-cold ethanol overnight at 4 °C. After being washed twice in PBS, cells were stained with a staining solution containing PI (50 μg/mL) and RNase A (200 μg/mL) for 30 min at room temperature. Cells were then harvested, washed, and suspended in PBS to a final concentration of 1 × 10^6^/mL and analyzed by BD FACS Aria flow cytometry (BD FACS Canto™, BD Biosciences).

### RNA sequencing and data analysis

To explore the molecular mechanisms why apatinib inhibited PC9GR but not PC9 cell proliferation, we conducted whole transcriptome assay by RNA sequencing (RNA-seq) on four groups of cells, parental PC9 cells (Group A), PC9 cells after 8 μM apatinib treatment for 48 h (Group B), PC9GR cells (Group C), and PC9GR (Group D) cells after 8 μM apatinib treatment for 48 h. Three replicates in each group were independently cultured and treated. At the end of the experiment, whole RNA was extracted and submitted to Annoroad Gene Technology Co., Ltd (Beijing, China) for sequencing. Library construction was performed following the manufacturer’s instructions and PolyA selection for enrichment of mRNAs was applied. Pair-end 150 base reads were sequenced on Illumina HiSeq 2500 instruments.

The raw data were processed through Annoroad pipeline for quality assessment and gene expression quantification. On average 47.5 million of reads ( ± 2 million) were generated with mapping efficiency of 97%. Pair-wise gene differential expression was performed using DESeq2 where genes with log2 fold change greater than 1 and q value (false discovery rate or FDR) < 0.05 were considered as differentially expressed genes (DEGs). Pathway or gene ontology (GO) enrichment analyses were conducted subsequently for these DEGs and those with false discovery rate (FDR) less than 0.05 were considered as significantly enriched. Heatmaps for the DEGs in selected pathways were generated by R package “heamap.3”. Pathway map was downloaded from KEGG (https://www.genome.jp/kegg/) where differentially expressed genes were highlighted with different colors.

### Quantitative real-time PCR

Total RNA was extracted from cells with TRIzol Reagent (Beyotime Institute of Biotechnology, China) and cDNA was synthesized using the PrimeScript RT Reagent Kit (Thermo Fisher Scientific, USA). Real-time RT-PCR (CFX 96, thermocycler, Bio-Rad, Hercules, CA) was performed to detect the expression of differentially expressed genes. GAPDH was used as a loading control.

### Protein expression by Western blotting

PC9GR cells were cultured with different concentrations of apatinib for 48 h, adherent cells were collected with trypsin, and then western blot analysis was performed. Cells samples were lysed on ice for 30 min in RIPA buffer (Beyotime Biotechnology) containing 1% protease inhibitor cocktail and phosphatase inhibitor cocktail (Biotool, Houston, TX, USA). The lysates were then centrifuged at 11,000*g* for 15 min at 4 °C. Then, the supernatant was mixed with 6 × loading buffer on a 5:1 scale, and the protein boiled in a water bath at 100 °C for 10 min. Equal amounts of cell lysates were separated by SDS-PAGE. After electrophoresis, the proteins were transferred onto a nitrocellulose membrane. The membrane was blocked with 5% non-fat milk in Tris-buffered saline and 0.05% Tween 20 (TBST) for 2 h at room temperature and then incubated with primary antibody at the appropriate dilutions overnight at 4 °C. The membranes were washed twice with TBST, 10 min at a time. They were then incubated with horseradish peroxidase (HRP)-conjugated secondary antibodies (Zhongshan Golden Bridge, Beijing, China) for 2 h at room temperature. The membranes were then washed three times with TBST and immunoreactive bands visualized using enhanced chemiluminescence (ECL) reagent (Pierce Fast Western Blot Kit, Thermo Scientific, Waltham, MA, USA) according to the manufacturer’s protocol. The relative expression ratios between the experimental and control groups were calculated based on density using the ImageJ software and the GAPDH signal as a reference.

### In vivo anti-tumor activity assessment in mouse xenograft models

Animals were treated according to protocols established by the ethics committee of Zhengzhou University and experiments were carried out in accordance with the approved guidelines and ethics committee of Zhengzhou University. Approximately 5 × 10^6^ PC9GR or PC9 cells were suspended in 0.15 mL PBS and injected subcutaneously into the back right flank region of 4–5 week-old, 18-g female BALB/c nude mice (Human SJA Laboratory Animal Co. Ltd, Hunan, China). Once the volumes reached approximately 100 mm^3^ (5 days after tumor inoculation), the mice were randomly assigned into four groups, 6 mice in each: (A) PC9GR cells, saline (negative control) group; (B) PC9GR cells, apatinib (100 mg/kg) treatment group; (C) PC9 cells, saline (negative control) group; and (D) PC9 cells, apatinib (100 mg/kg) treatment group. The treatment groups received oral administration of apatinib once a day for a total of 21 days. The body weight and the tumor volume (V) were recorded every 2 days. The V was calculated as 0.5 × length × width^2^. At the end of 21 days, the mice were sacrificed and the tumors were isolated for measurement and weighing. Immunohistochemistry (IHC) staining was performed for protein expression of VEGFR2, phosphorylated VEGFR2 and CD31 on tumor blocks. All of the antibodies, reactivity, and conditions were tested in positive control slides. The primary antibodies were visualized with the corresponding biotinylated antibody coupled to streptavidin–peroxidase complex. Histologic micrographs were taken using a Leica DM200 microscope. Image-Pro Plus 6.0 software was used to quantify the expression of indicated proteins.

### Statistical analysis

All data except RNA-seq were analyzed using GraphPad Prism (version 5.0, San Diego, CA, USA), which include one-way analysis of variance (ANOVA) or *t* test. The results were presented as the mean ± SD. A p value less than 0.05 was considered to be statistically significant among groups.

## Results

### Establishment of PC9 gefitinib-resistant cells mimic to clinical observation

**Fig. 1:**
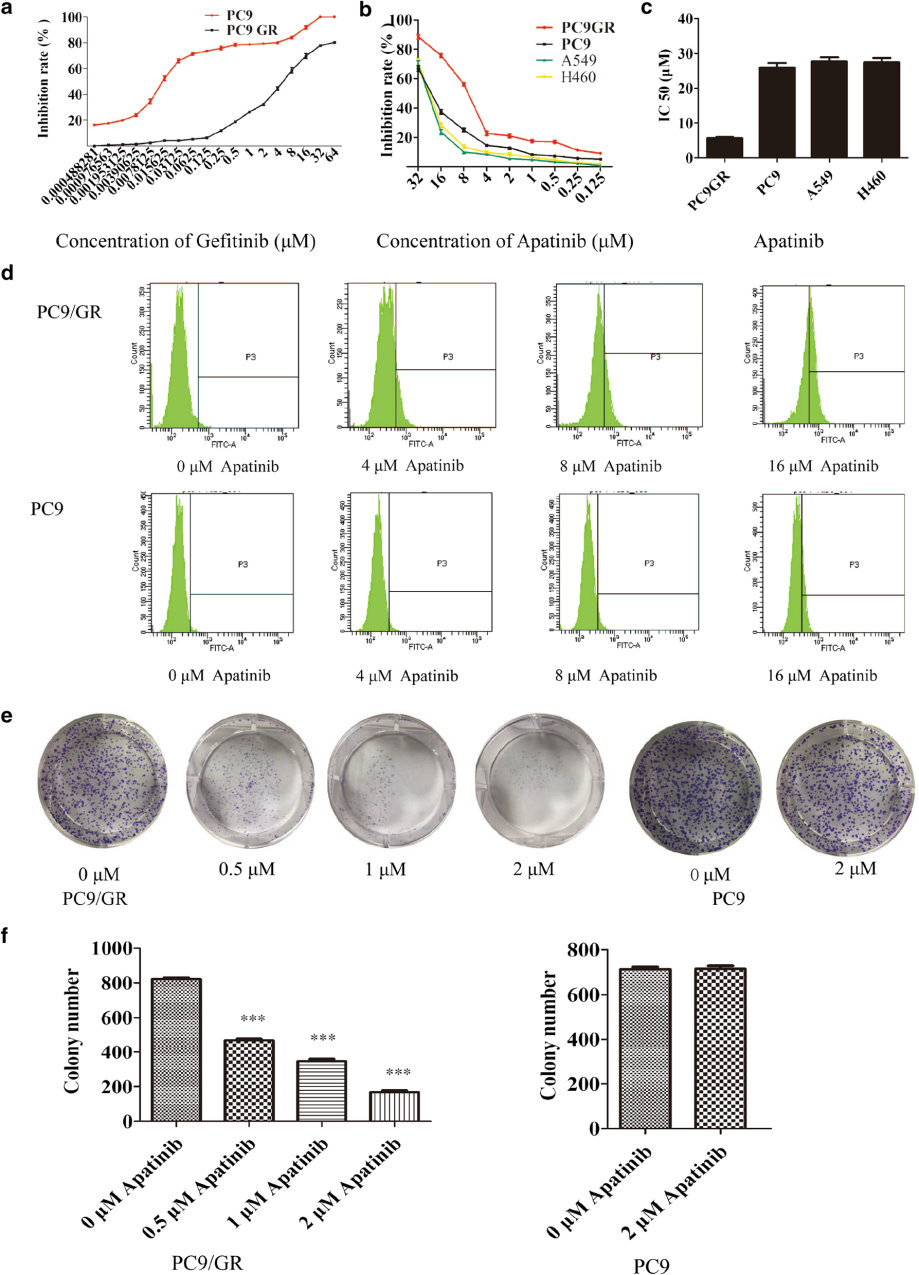
Apatinib inhibits the growth of PC9GR cells in vitro.** a** The inhibition rate, as measured by CCK-8 after PC9 and PC9GR cell treated with different concentrations of gefitinib. PC9GR cells have a significantly increased IC50 and demonstrate strong resistance to gefitinib. **b** Cell viability of PC9, A549, H460, and PC9GR cells after treatment with different concentrations of apatinib, determined by CCK8 assay at the indicated time. PC9GR cells are most sensitive than other cells. **c** The IC_50_ values of PC9, A549, H460, and PC9GR cells treated with apatinib where PC9GR cells are significantly lower. **d** Results of flow cytometry for PC9GR and PC9 cells treated with different concentrations of apatinib for 24 h where increasing fluorescence intensities were seen with increasing concentrations in PC9GR cells but not in the PC9 cells. **e** Colony formation of PC9GR and PC9 cells at different concentrations of apatinib. Colony numbers are decreasing along with increasing apatinib concentrations in PC9GR cells but there are no changes in PC9 cells. **f** Bar plots for numbers of colonies from PC9GR and PC9 cells at different concentrations of apatinib. ***Significantly different (p < 0.001) from control cells

After 1 year of gefitinib treatment, we successfully established a gefitinib-resistant NSCLC cell line PC9GR from the gefitinib-sensitive PC9 cell line, which is derived from lung adenocarcinoma with a typical EGFR mutation on exon19 (15 base deletion). The IC_50_ value for gefitinib in PC9GR cells was 5.311 ± 0.455 μM, which is a 265-fold higher than that in PC9 cells (0.020 ± 0.003 μM) (Fig. 1a). Morphologically, PC9GR cells were smaller and rounder than PC9 parental cells. They were easier to grow and form a denser layer on culture, indications of higher proliferation ability. From RNA-seq of these cell lines, we validated that both PC9 and PC9GR cells had 15-base deletion on exon 19 of EGFR. More noticeably, the PC9GR cells obtained a newly developed T790M mutation not present in their parental PC9 cells. This mutation is known to be one of the main reasons in clinic for patients who develop TKI resistance after treatment [19–21].

### Apatinib strongly inhibited the growth of PC9GR cells but not other lung cancer cell lines

We somewhat accidently found that apatinib had much stronger inhibitory effects on PC9GR cells than on its parental sensitive and other lung cancer cell lines. We used the CCK8 method to determine the inhibition rates of 9 different concentrations of apatinib incubated for 72 h along with its parental PC9 and two other lung caner cell lines A549 and H460. As shown in Fig. 1b, apatinib showed a nearly fivefold stronger inhibitory effect on PC9GR cells (an IC_50_ value of 5.92 ± 0.11 μM) than on lung cancer cell lines PC9, A549, and H460 (IC_50_ values were 25.99 ± 1.76 μM, 27.72 ± 1.71 μM, and 27.44 ± 1.83 μM, respectively). The IC_50_ value of PC9 parental cells was four times higher than that of PC9GR cells (Fig. 1c).

To determine whether the decreased cell viability after apatinib treatment was caused by increased cell apoptosis or reduced cell proliferation, we observed the cells in a Petri dish. At a concentration of 5.92 μM (IC_50_), the PC9GR cells maintained the similar morphology as the untreated cells and did not have any noticeable apoptosis signs; however, its cell population showed dramatic depletion. This was not observed for the PC9 cells after the same treatment where neither morphology nor cell number had any change.

We further examined the anti-proliferation effect of apatinib on PC9GR through CFDA-SE fluorescence assay. After treatment with apatinib at 4 different concentrations (0, 4, 8, and 16 μM) for 72 h, PC9 and PC9GR cell proliferation rates were measured. The cell division suppression was found to be much more obvious in PC9GR cells than in PC9 cells, where gradually increased fluorescence intensities with increasing concentrations were clearly observed in PC9GR cells but not in the PC9 cells (Fig. 1d).

We next used colony formation assays to determine whether apatinib could affect proliferative capacity of single cells. PC9 and PC9GR cells were seeded in a 6-well plate at 2000 cells/per well and treated with apatinib at four concentrations (0, 0.5, 1, and 2 μM) for 7 days. The colony formations were then photographed and counted using ImageJ software. As shown in Fig. 1e, f, the numbers of colonies in the PC9GR cell group were significantly reduced with the increase of apatinib concentrations. In contrast, apatinib did not inhibit PC9 cell colony formation, even at 2 μM. Taken together, these results showed that apatinib more specifically and effectively inhibited the proliferation of PC9GR cells.

### Apatinib induced cell cycle arrest but not apoptosis in PC9GR cells

**Fig. 2:**
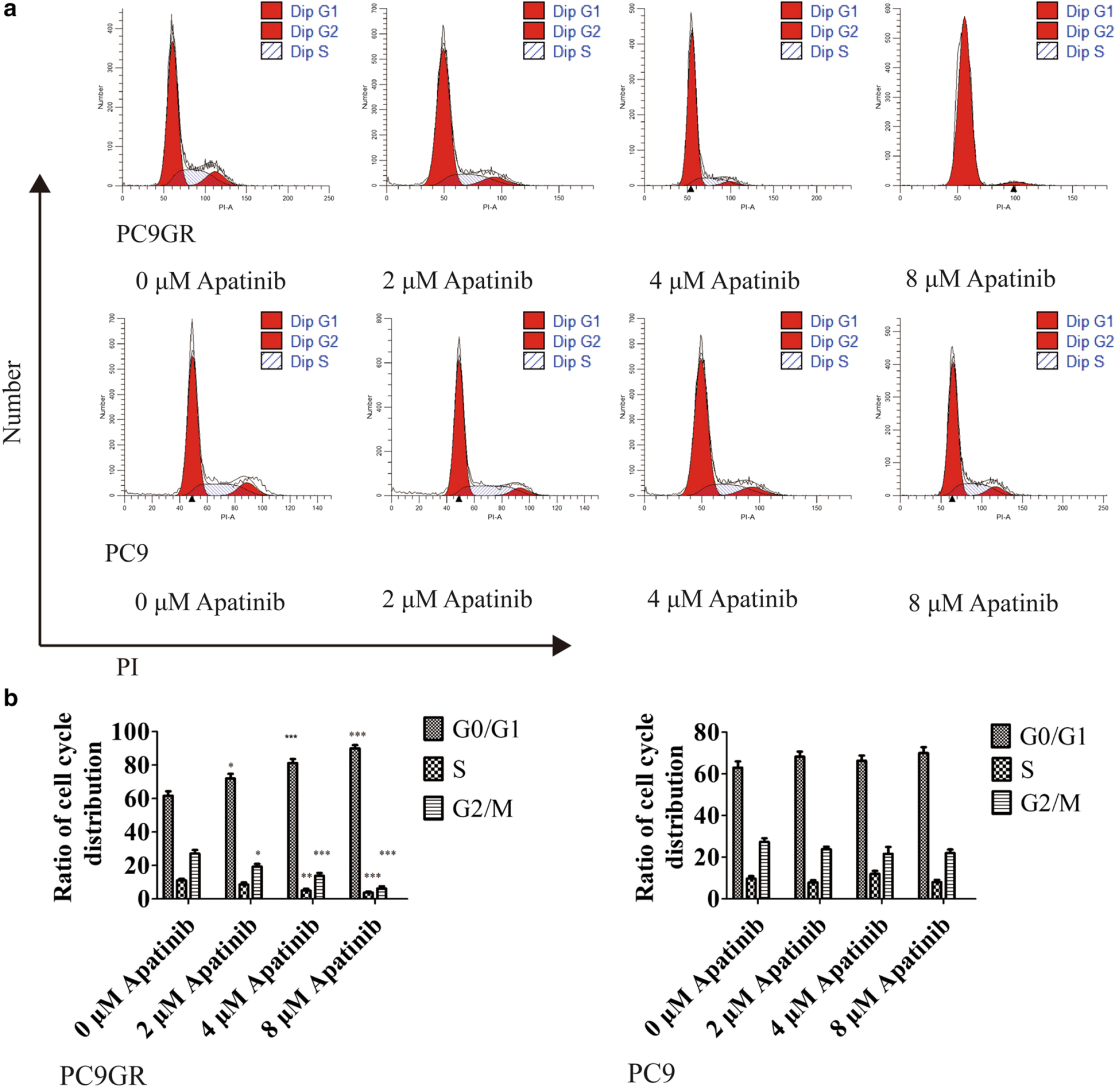
Apatinib induces cell cycle arrest in PC9GR cells. **a** Number of PC9GR and PC9 cells in different stages of cell cycles after treatment with different concentrations of apatinib for 48 h. **b** Bar plots of cell distribution in different cell cycles as measured using flow cytometry. There were at least three independent experiment replicates at each condition. *p < 0.05, **p < 0.01, ***p < 0.001 compared with control using one-way ANOVA

**Fig. 3:**
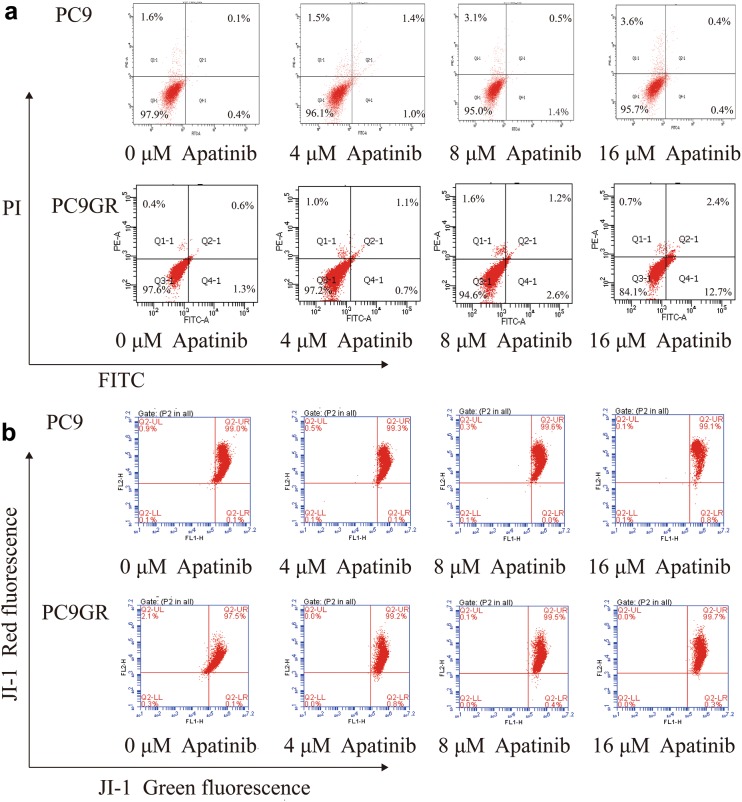
Apatinib does not lead to significant apoptosis in PC9GR cells. **a** The proportions of PC9GR and PC9 cell apoptosis after treatment with apatinib for 48 h, determined by Annexin V-FITC and PI staining. No significant differences were seen at lower concentration ( < 8 μM) across cell lines at the same concentration or in the same cell lines between different concentration although slightly increased late apoptosis was observed in PC9GR cells at 16 μM. **b** The mitochondrial membrane potential of PC9GR and PC9 cells treated with apatinib for 24 h, measured by flow cytometry with JC-1 staining. No obvious differences were observed between PC9 and PC9GR cells

As demonstrated above, apatinib effectively inhibited the growth of PC9GR cells in a concentration-dependent manner. To further explore where the inhibition occurred, we performed cell cycle analysis. After treatment with different concentrations (0, 2, 4, and 8 μM) of apatinib for 48 h, PC9 and PC9GR cells were fixed and stained with PI for flow cytometry analysis. As shown in Fig. 2a, b, the arrest of the PC9GR cell cycle happened in the G1 phase and showed apatinib-concentration dependent. More specifically, with treatment at the highest concentration (8 μM) of apatinib, the percentage of cells in the G1 phase was 90.03% in the PC9GR cells, about 28% more than that of the control group. However, we did not find any significant cell cycle distribution change in PC9 cells with the same treatment concentrations. Next, we performed flow cytometric analysis of PC9 and PC9GR cells using Annexin V-FITC and PI double staining after incubation with apatinib at different concentrations (0, 4, 8, and 16 μM) for 48 h. As shown in Fig. 3a, there was no much difference of late apoptotic cells between PC9GR and PC9 cells at lower concentrations (4 and 8 μM), although at 16 μM, the late apoptotic cells in PC9GR were slightly higher at 12.7% (vs. 0.4% in PC9 cells, > 30% considered as obvious apoptosis). Apatinib did not induce noticeable apoptosis in PC9 cells in any of concentrations applied. Apoptosis is associated with mitochondrial dysfunction, as indicated by change of mitochondrial membrane permeability, which leads to a loss of MMP and activation of downstream caspases. Therefore, we measured MMP using JC-1 dye to evaluate apatinib-induced mitochondrial dysfunction. As illustrated in Fig. 3b, apatinib did not increase green fluorescence intensity in both PC9 and PC9GR cells, demonstrating that apatinib-induced JC-1 dye did not migrate from mitochondria to the cytoplasm. Additionally, no significant changes in MMP, represented by green/red fluorescence intensity, were observed by flow cytometry assay. All these suggested that although apatinib could induce apoptosis of PC9GR cells slightly, mitochondrial function was not significantly compromised and apoptosis was not the main reason for the inhibition of PC9GR cell proliferation by apatinib.

### Transcriptome provided clues of molecular mechanisms of PC9GR cells responsive to apatinib

**Fig. 4:**
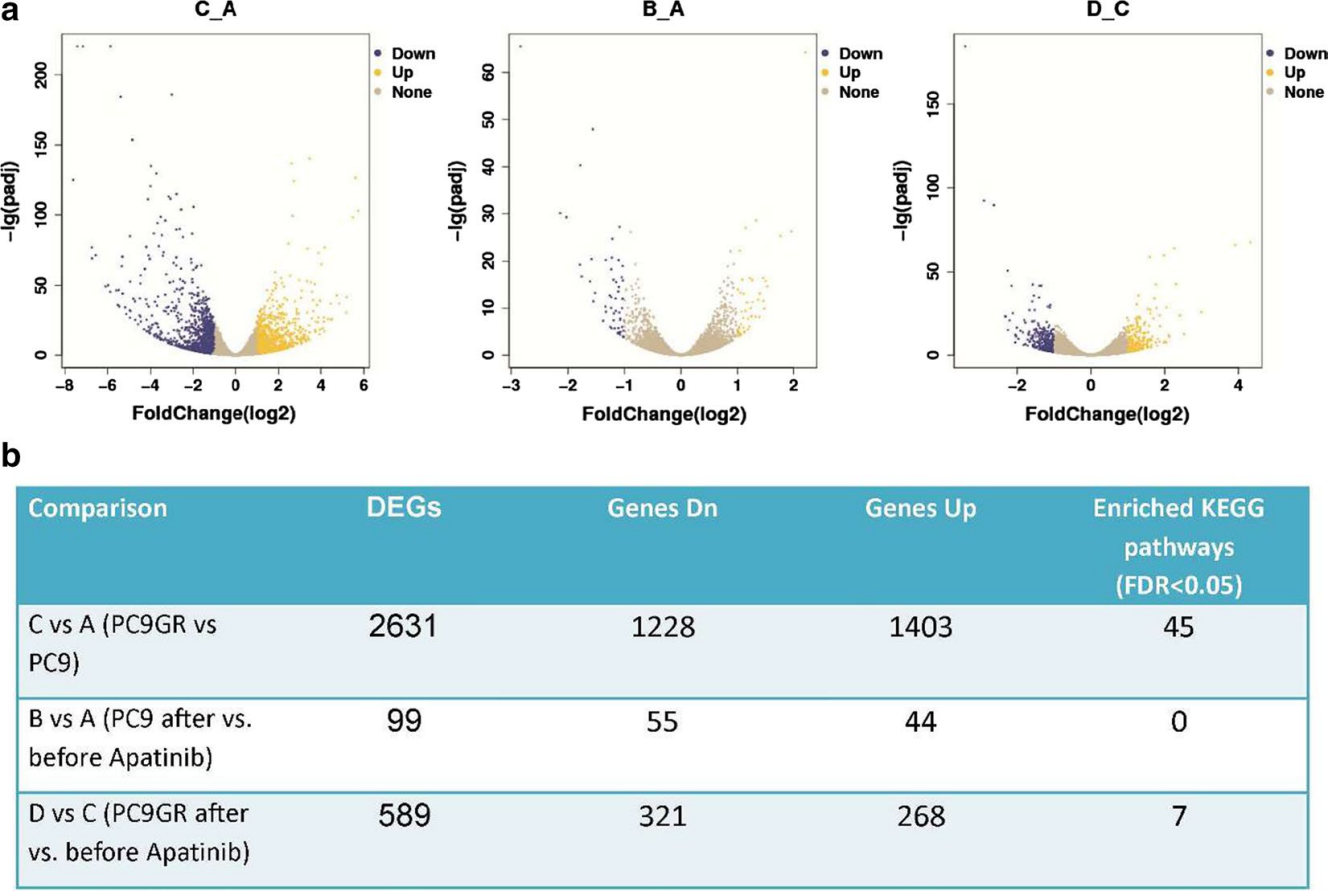
Differentially expressed genes among different groups of PC9/PC9GR cells from RNA sequencing.** a** Vocano plot of differentially expressed genes (DEGs) between C vs. A, B vs. A, and D vs. C group. X-asix represents log2 fold change, blue for down expression and yellow for up expression. **b** Summary of DEGs and enriched pathways

**Fig. 5:**
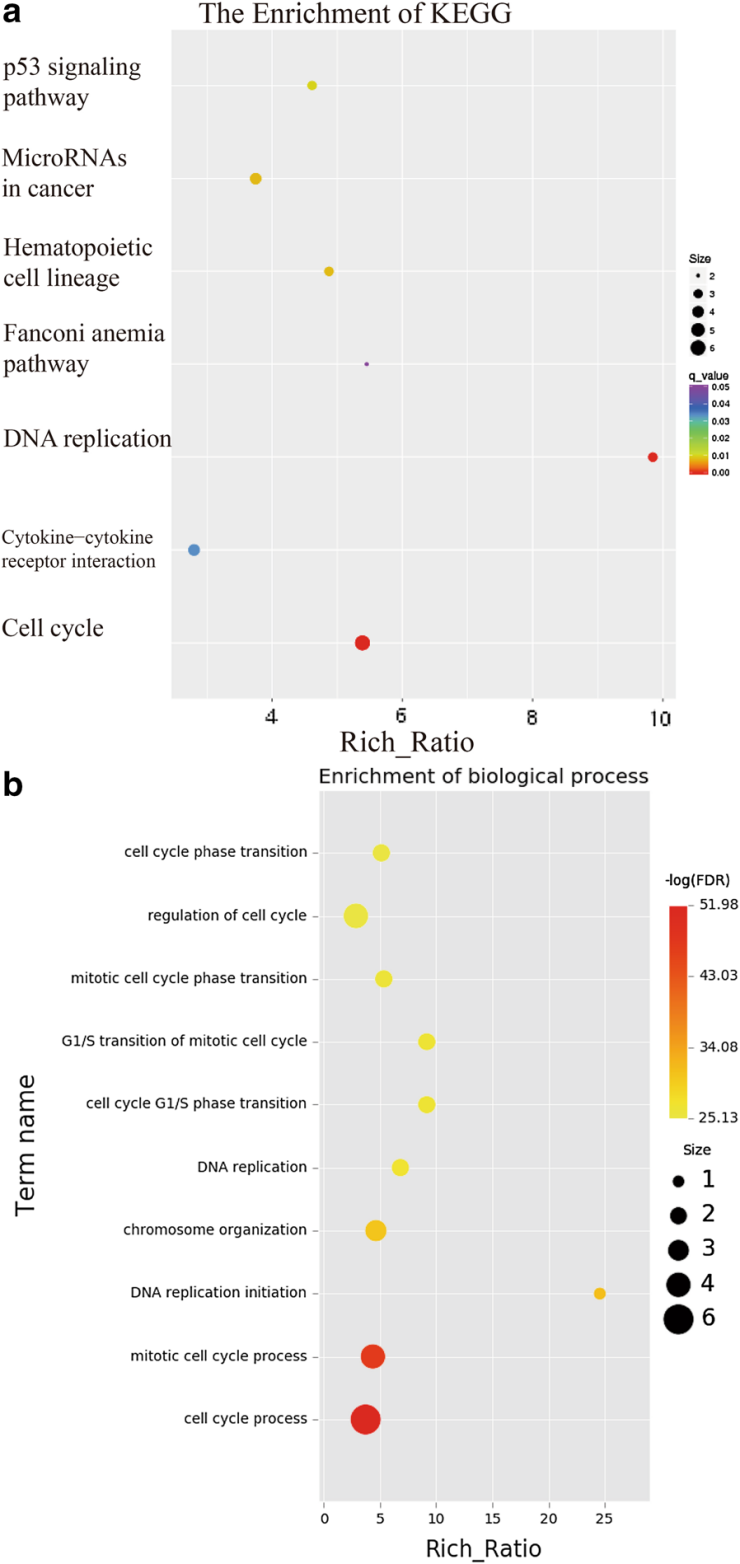
Pathway enrichment for PC9GR cells after treatment with apatinib. **a** Enriched KEGG pathways. Cell cycle and DNA replication were mostly enriched by p value. **b** Gene ontology (GO) analysis of enriched biological processes. The two most significant ones are cell cycle and mitotic cell cycle process. network maps

**Fig. 6:**
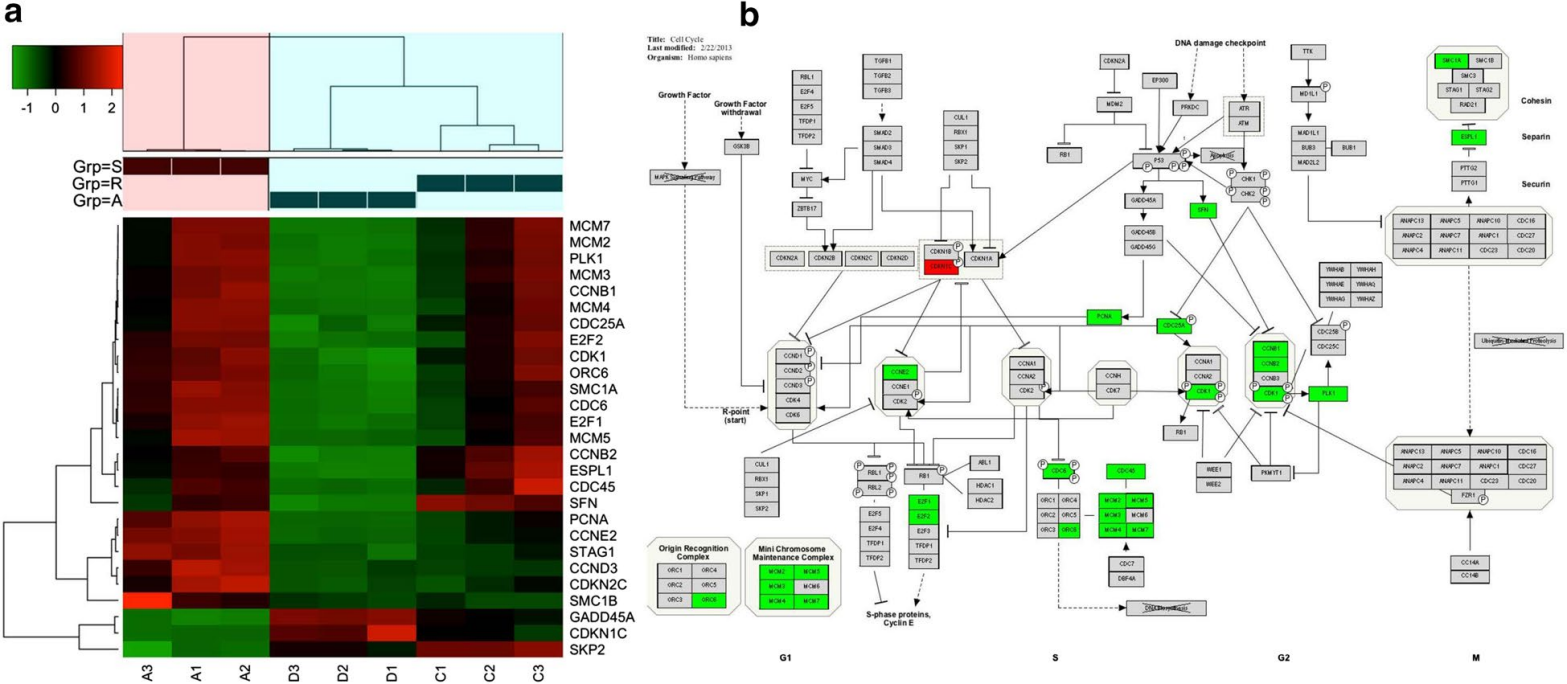
Differentially expressed genes in cell cycle pathway. **a** Heatmap for differentially expressed genes in any of A, C, D group comparisons. The changed genes by comparison of PC9 and apatinib-treated PC9 cells. **b** DEGs in cell cycle pathway after treatment of apatinib in PC9GR cells. Only CCKN1C is up regulated and all others are down regulated

To get a picture of molecular underpins why PC9GR cells were highly responsive to apatinib, we obtained the transcriptome data for 4 groups of cells, parental PC9 cells (Group A), PC9 cells after apatinib treatment (Group B), PC9GR cells (Group C), and PC9GR (Group D) cells after apatinib treatment. We focused our analysis between group B and A, D and C, and C and A, representing genes changed after apatinib treatment for sensitive cells (PC9), resistance cells (PC9GR), and before treatment for PC9GR cells (resistance related genes). We reasoned that genes that were changed after treatment were the targets and downstream effects of apatinib and the genes that were different between PC9GR and PC9 without treatment rendered the different responsiveness to apatinib. As expected, there were only 99 genes differentially expressed between group B and A (Fig. 4a, b) (Additional file 1), suggesting the treatment did not have much impact on PC 9 sensitive cells. No pathway enrichment for these genes was observed. On the contrary, 589 genes were differentially expressed between group D and group C (Additional file 2), the TKI resistant PC9GR cells after apatinib treatment (vs. untreated cells). These genes were associated with 7 enriched KEGG pathways (Fig. 5a) and 10 enriched GO biological processes (Fig. 5b). Noted is that the top 2 enriched pathways are Cell Cycle and DNA replication and among 21 DEGs in Cell Cycle and 10 DEGs in DNA replication all but one (CDKN1C) in Cell Cycle were down-regulated after apatinib treatment (Fig. 6a, b). CDKN1C is a known negative regulator of cell proliferation and its increase indicated its role in reduced activity of cell cycle. These transcriptome data were in line with our previous observations that cell cycle was significantly inhibited in PC9GR cells after apatinib treatment. To validate the results from RNA-Seq, 5 differentially expressed and 1 not differentially expressed genes in cell cycle pathway were selected for qRT-PCR analysis using GAPDH as the internal reference gene. Among these genes, CDKN1C was verified to be up-regulated in apatinib-treated PC9GR cells whereas the other four (CDK2, CCNE2, E2F1 and E2F2) were confirmed as down-regulated. CDKN1C was also found without significant change (Additional file 3).

In addition to cell cycle, we next explored the important but harder question why PC9GR cells were resistant to gefitinib but more sensitive to apatinib treatment. For this we compared PC9GR to PC9 cells and found over 2600 DEGs (1403 up-regulated and 1228 down-regulated) (Additional file 4). KEGG pathway enrichment analysis for these genes revealed 45 enriched pathways (Additional file 5) and the top signal pathways included Cytokine-cytokine reception interaction, cAMP signaling pathway, Rap1 signaling pathway, and MAPK signaling pathway. The large numbers of changed genes and pathways suggested that the resistant cells had very different transcription, signaling and metabolic programs.

As apatinib primarily targets VEGF pathway, we were interested in knowing if there were any genes changed in this pathway between PC9GR cells and PC9 cells and PC9GR before and after apatinib treatment. There were 6 genes differentially expressed between resistant PC9GR and PC9 cells, all but one (PIK3R3) were up expressed (Additional file 6), indication of a more active state. Two genes (PLCG2 and SHC2) were further increased after apatinib treatment. However, this pathway was not significantly enriched for either comparison in pathway analysis because of the limited numbers of changed genes.

### Apatinib arrested the cell cycle by regulating the p57/cyclin-E2 /Rb signaling pathway as evidenced by protein expression

Multiple pieces of evidence presented above clearly showed that low concentrations of apatinib could arrest PC9GR cells in the G1 phase through interrupting cell cycle and DNA replication. As final acting molecules are proteins, we examined protein expression of well-known cell cycle regulators associated with G1 phase arrest.

**Fig. 7:**
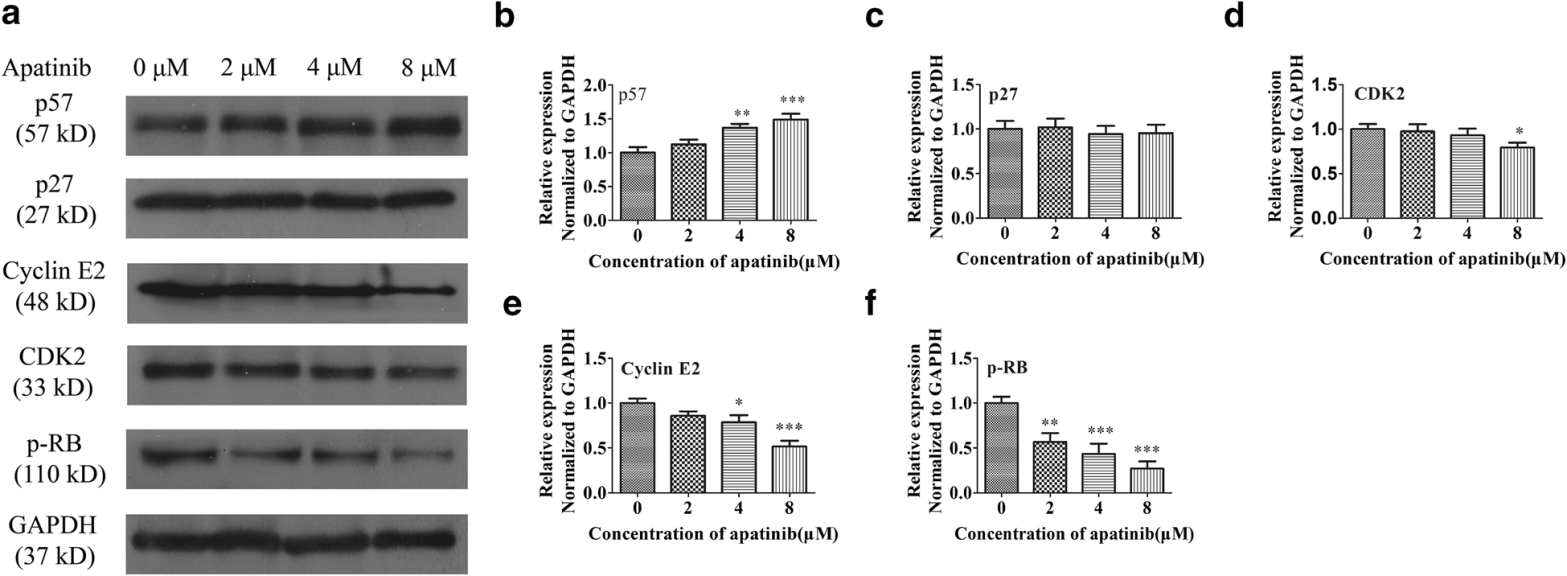
Protein expression by Western blot for selected proteins in p57/cyclin-E2/Rb signaling pathway in PC9GR cells. **a** Western blot showing reduced expression levels of cyclin E2, CDK2, pRb and increased expression level of p57 in apatinib-treated cells. **b**–**f** Statistical analysis of p27, p57, Cyclin E2, CDK2, and p-Rb expression levels. *p < 0.05, **p < 0.01, ***p < 0.001 compared to control group with 0 concentration

As shown in Fig. 7, apatinib increased p57 (CDKN1C) but reduced CDK2 and cyclin E2 (CCNE2) protein levels in a dose-dependent manner, all consistent with mRNA expression from RNA-seq and suggesting that apatinib arrested cells in the G1 phase through activating the p57 protein and inhibiting the function of the CDK2/cyclin E2, G1 and S phase kinase complexes. The expression of pRb (RB1 gene, also reduced in RNA-seq) was also decreased after apatinib treatment. These results further confirmed from protein expression of well-know cell cycle arrest players that apatinib arrested the cell cycle at the G1 phase by regulating the p57/cyclin E2/Rb signaling pathways.

### Apatinib inhibited the tumor growth of PC9GR cell xenograft in mice

**Fig. 8:**
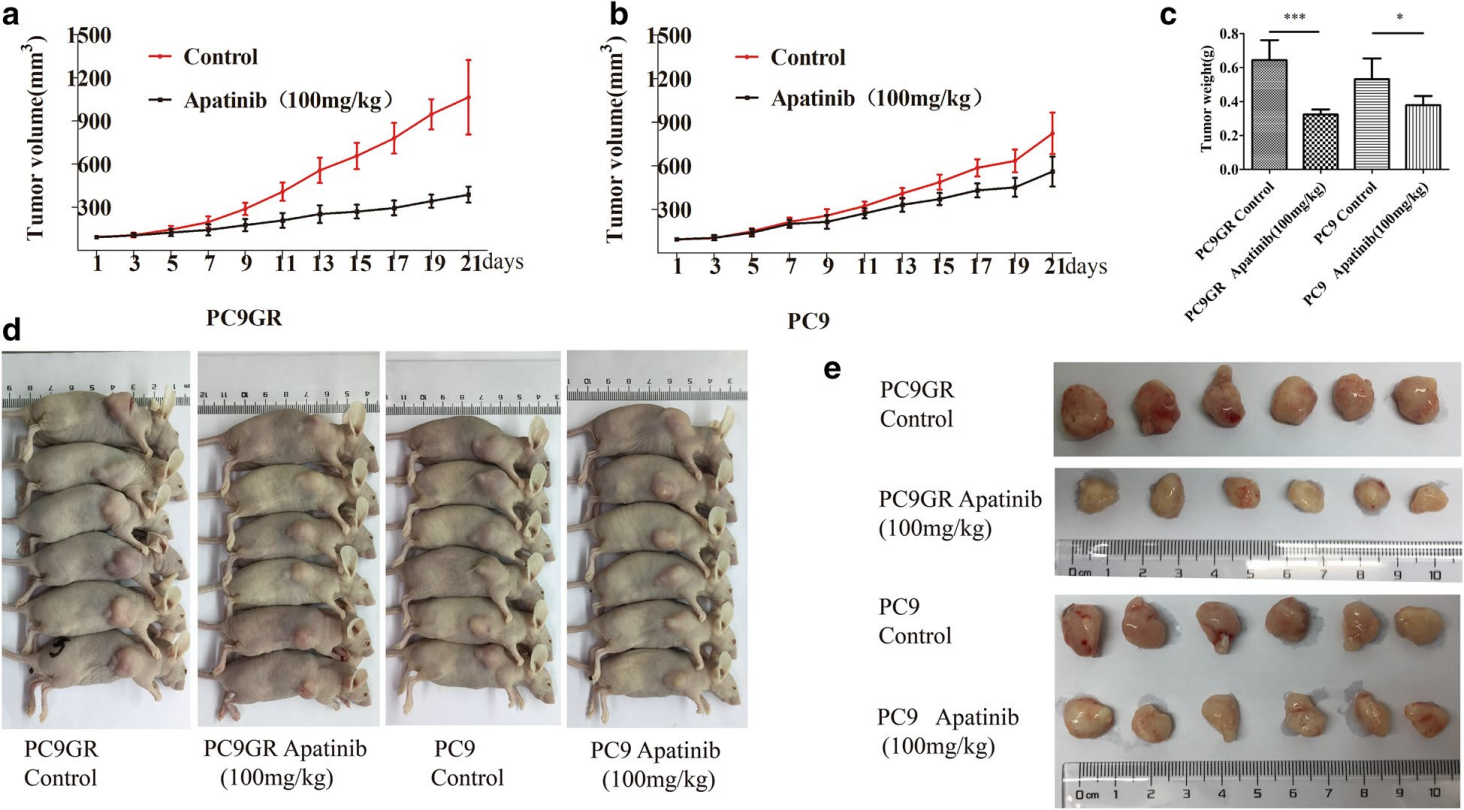
Apatinib inhibits the tumor growth of PC9GR cell xenograft in mice. **a**, **b** Volume changes of tumors in mice bearing PC9GR and PC9 cells, respectively over 21 days by administration of 100 mg/kg apatinib daily. **c** Tumor weight changes in mice bearing PC9GR or PC9 cells after treatment where PC9GR tumors shrunk the most. **d** Photographs of mice in each group and **e** photographs of tumors in each group. Each group has 6 mice or tumors. Each value represents a group mean ± standard deviation. *p < 0.05, **p < 0.01, ***p < 0.001 compared with the control group using the *t* test

To evaluate if apatinib has the similar anti-cancer effect on gefitinib-resistant tumor in vivo, we established Xenograft models by subcutaneously implanting PC9 or PC9GR cells. Once the tumors were established ( ~ 100 mm^3^ after 5 days of implantation) in 12 mice per cell line, the mice were randomly assigned into apatinib treatment and placebo control group (within cell line, six mice per group, a total of 4 groups). Apatinib was administered to the treatment groups orally at the dose of 100 mg/kg, once a day for a total of 21 days and saline was used as a negative control for the control groups. As shown in Fig. 8a–c, apatinib inhibited tumor growth for both PC9 and PC9GR established tumor grafts compared to their respective control group; however, its anti-tumor effect was much stronger in the PC9GR grafts than the PC9 grafts as measured by tumor volume and weight. Specifically, at the end of the experiment, the mean volume and weight of the tumors established from PC9GR treated with saline were 1065 mm^3^ and 0.644 g, respectively; however these measures in the group treated with apatinib were 388 mm^3^ (p < 0.01) and 0.323 g (p < 0.01), about 63.5% and 49.8% reduction, respectively. For the tumor grafts from PC9 cells, the volume and weight in the control group were 824 mm^3^ and 0.532 g, respectively, compared to 561 mm^3^ (p < 0.01) and 0.379 g (p < 0.05) for the group with apatinib treatment (31.9% and 28.7% reduction, Fig. 8d, e). Although oral administration of apatinib also reduced the volume and weight of subcutaneous PC9 xenograft tumors in nude mice, the therapeutic effect was significantly weaker than in the PC9GR cells. Noted also is that tumor grafts from PC9GR cells were larger than those from PC9 cells (in the untreated control groups), suggesting resistant cells were more aggressive and faster growing.

**Fig. 9:**
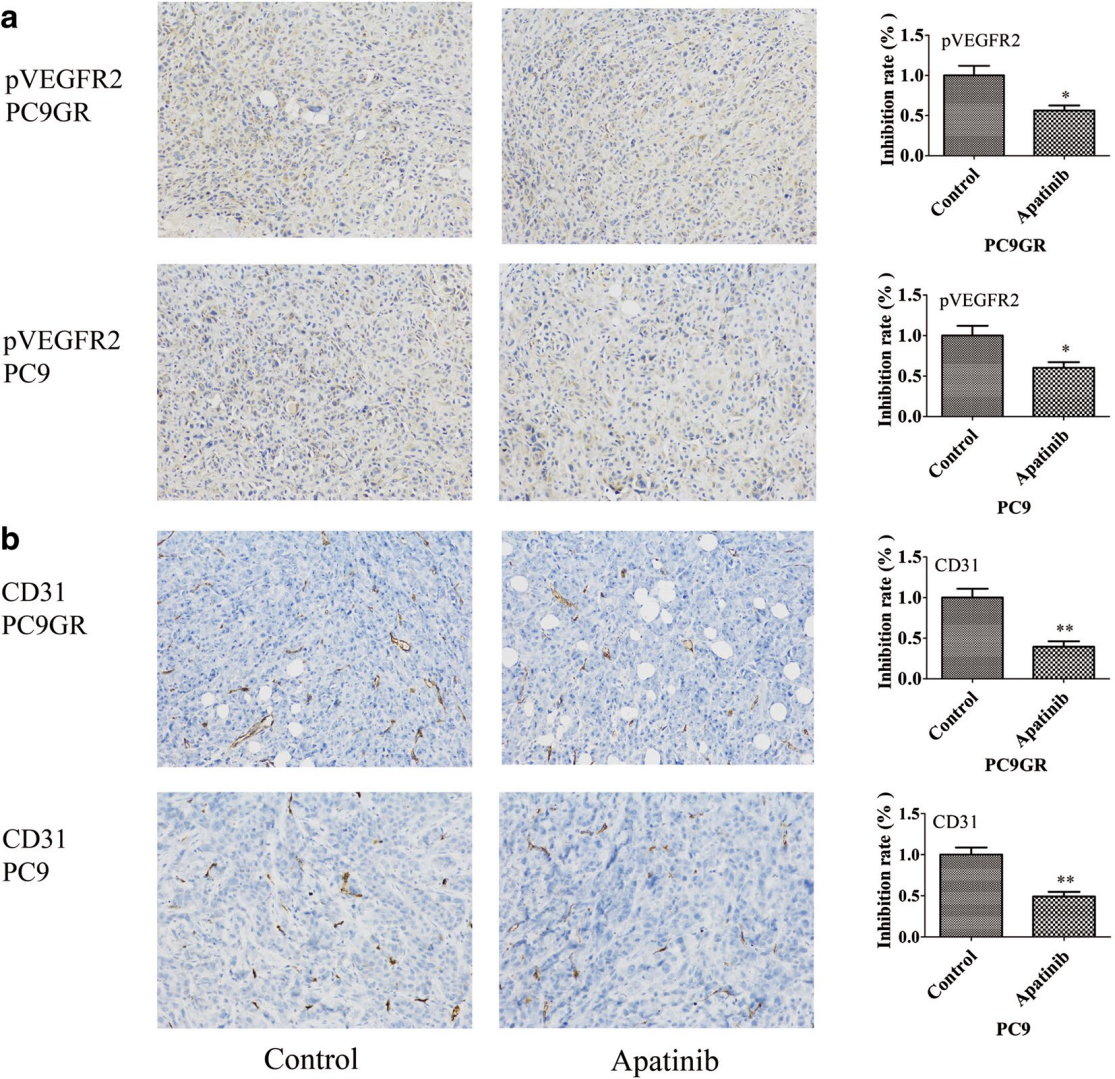
Effects of apatinib on the expression of pVEGFR2 and CD31 in xenograft models. **a** Immunohistochemical staining for pVEGFR2 and **b** CD31 in tumor tissues obtained from the control and apatinib-treated mice. The bar plot value represents the mean ± standard deviation of at least three independent replicates. *p < 0.05, **p < 0.01 compared with control, using the *t* test

Apatinib is a highly selective inhibitor of VEGFR2 so we were interested whether the treatment affected its expression and/or phosphorylation and blood vessel formation in tumor grafts between treated and untreated mice. To this end, we used IHC to measure the protein expression of VEGFR2, CD31 (a blood vessel endothelial marker), and the phosphorylated VEGFR2 on tumor graft blocks. No significant difference was found for VEGFR2 positivity between apatinib-treated and control groups, in both PC9GR and PC9 derived xenografts (p > 0.05, Fig. 9a, left panel). However, the phosphorylation level of VEGFR2 was significantly reduced in apatinib treated group (Fig. 9a, right panel). The microvessels as indicated CD31 were fewer in apatinib treated tumors (Fig. 9b).

During the 21 days of apatinib administration, we did not observe any significant differences among the 4 groups in terms of body weight or abnormal behaviors. No mouse died in any group and there were no significant changes in the weight, color and texture of vital organs, including the liver, kidney, brain, heart, lung, spleen, and thymus between treatment and control groups. These results demonstrated that apatinib was well tolerated and did not have noticeable toxicities at a dose of 100 mg/kg.

## Discussion

In this study, we report that apatinib did not have anti-proliferative effects on lung cancer cell lines, PC9, A549, and H460. However, it significantly inhibited the proliferation of gefitinib resistant PC9GR cells. We demonstrate that this inhibition was through arresting cells in the G1 phase of the cell cycle. To the best of our knowledge, this has not been reported before and may provide an alternative option to treat patients after developing EGFR-TKI resistance after usage.

Apatinib is a novel small-molecule anti-angiogenic agent and a TKI that selectively inhibits VEGFR2. It can block the signal transduction of VEGF and its receptor, strongly inhibiting angiogenesis and exerting anti-tumor effects [29], which has been used clinically as an anti-angiogenic drug. Based on registered phase I, II, and III clinical trials, apatinib demonstrated a desirable therapeutic effect with a higher survival rate in patients with advanced gastric cancer who failed standard chemotherapy [30]. Our data in mouse xenografts showed it indeed reduced the phosphorylation of VEGFR2 without increasing its expression and affected microvessel formation. What our data uniquely showed is that apatinib only had strong inhibition effects on cell cycle for the cells with gefitinib resistance but not their parental sensitive cells. The T790M mutation and dramatically changed gene expression programs may explain the difference as demonstrated in RNA-seq and protein expression assays for p57, CDK2, cyclin E2, and pRb. The cell cycle arrest effect was reported previously in hepatocellular carcinoma cells [37] and cervical cancer [38], suggesting cell cycle is an important target of apatinib. Our data also showed that apatinib had no much impact on apoptosis in PC9GR at lower concentrations although it had a minor impact at higher concentration. Other studies indeed report it may have an apoptotic effect on cancer cells such as cervical cancer [38] and osteosarcoma [39] and this effect may be cancer cells specific.

From RNA-seq data, we found that many pathways such as MAPK, focal adhesion, and PI3K-Akt signaling pathways changed after gefitinib resistance, consistent with previous studies. After apatinib treatment, there were few changed genes for PC9 cells. On the contrary, many genes were changed for PC9GR cells after the treatment and these genes were strongly enriched in cell cycle process, DNA replication initiation, cell cycle G1/S phase transition, and G1/S transition of the mitotic cell cycle. These observations clearly show that after development of gefitinib resistance the dramatically changed pathways in PC9GR cells render new sensitive targets for apatinib and cell cycle may be an important one. Apatinib’s primary target, VEGF pathway, had some changed genes but the pathway itself was not significantly enriched, which may be explained by the fact that VEGF pathway may be mainly active in endothelial cells, not cancer cells. However, it may play an important role as shown in our mouse xenograft models.

Apatinib has been used in clinical trial settings for several cancers. It is well tolerated and has shown clinical benefits [40–43]. It is generally administrated alone or in combination with other drugs. However, its usage for patients after targeted TKI resistance with EGFR mutation is indicated and further investigation is warranted.

## Conclusion

In conclusion, our study shows that apatinib has a strong therapeutic effect on gefitinib resistant cells/tumors and this effect is mainly mediated through direct cell cycle arrest effects. Apatinib may be indicated for patients with lung adenocarcinoma with acquired resistance after targeted TKI treatment.

## Additional files

**Additional file 1.** Differentially expressed genes in PC9 cells before and after treatment with apatinib.

**Additional file 2.** Differentially expressed genes in PC9GR cells after treatment with apatinib.

**Additional file 3.** mRNA expression for selected cell cycle genes in PC9GR cells before and after treatment of apatinib through RT-PCR. The experiments were performed in triplicate, and the data were presented as mean ± SD, ***p < 0.0001 vs. control group.

**Additional file 4.** Differentially expressed genes in PC9 cells and PC9GR cells.

**Additional file 5.** Significantly enriched pathways for differentially expressed genes between PC9GR cells and PC9 cells.

**Additional file 6.** Differentially expressed genes in VEGF pathway. A. a heat map for DEGs in any of C vs. A and D vs. C comparison. B. DEGs highlighted in VEGF pathway, red for up regulation and green for down regulation.

## Abbreviations

IC_50_: half maximal inhibitory concentration
TKIs: tyrosine kinase inhibitors
EGFR: targeting epidermal growth factor receptor
GR: gefitinib-resistant cells
NSCLC: non-small cell lung cancer
JAK/STAT: Janus kinase/signal transducers and activators of transcription pathway
PI3K: phosphoinositol 3-kinase/Akt pathway
MAPK: mitogen-activated protein kinase pathway
VEGFR2: vascular endothelial growth factor receptor 2
GO: gene ontology
