# Supplementary figures and images for: Apatinib preferentially inhibits PC9 gefitinib-resistant cancer cells by inducing cell cycle arrest and inhibiting VEGFR signaling pathway
Source: Cancer Cell Int. 2019 May 2;19:117. doi: 10.1186/s12935-019-0836-8 (PMC6498592; doi:10.1186/s12935-019-0836-8)

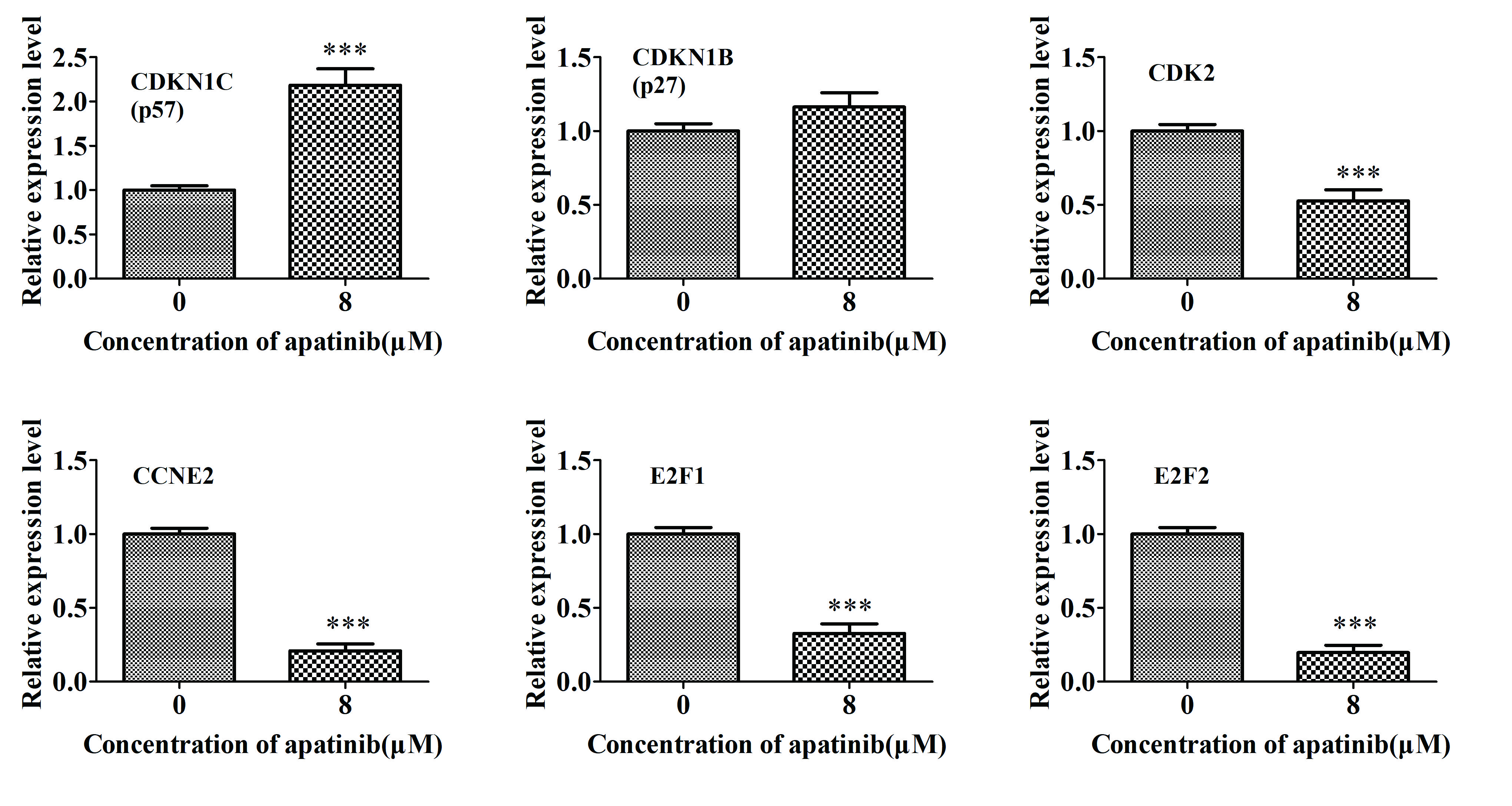

Supplement: Supplementary file 3 — Additional file 3. mRNA expression for selected cell cycle genes in PC9GR cells before and after treatment of apatinib through RT-PCR. The experiments were performed in triplicate, and the data were presented as mean ± SD, ***p < 0.0001 vs. control group. [file 12935_2019_836_MOESM3_ESM.jpg]

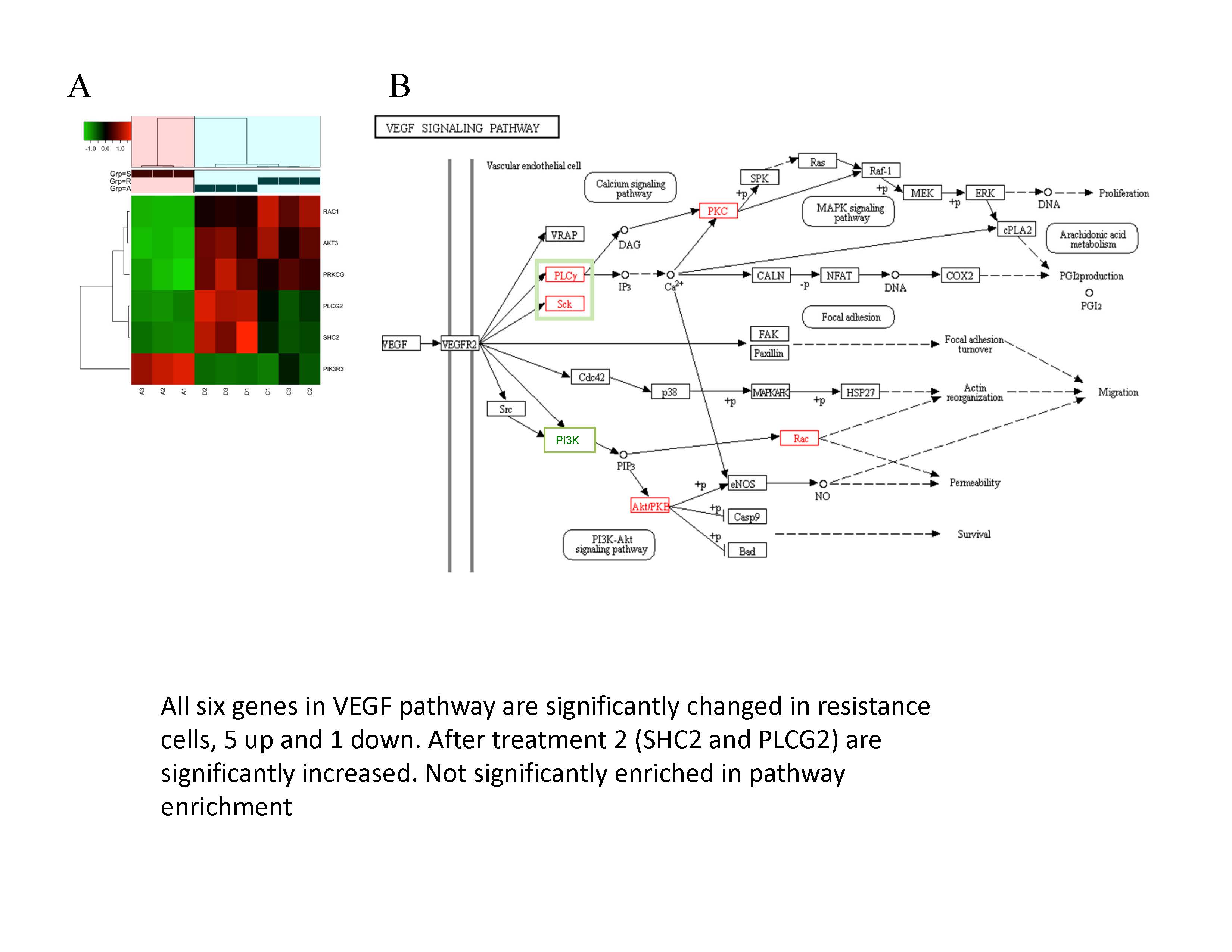

Supplement: Supplementary file 6 — Additional file 6. Differentially expressed genes in VEGF pathway. A. a heat map for DEGs in any of C vs. A and D vs. C comparison. B. DEGs highlighted in VEGF pathway, red for up regulation and green for down regulation. [file 12935_2019_836_MOESM6_ESM.jpg]
